# Using outcomes data to justify instituting new technology: a single institution’s experience

**DOI:** 10.1007/s00464-017-6001-3

**Published:** 2017-12-22

**Authors:** P. M. Starker, B. Chinn

**Affiliations:** 1 Overlook Medical Center, Columbia University College of Physicians and Surgeons, New York, NY USA; 2 Division of Colon and Rectal Surgery, Overlook Medical Center, Robert Wood Johnson Medical School, New Brunswick, NJ USA; 3 11 Overlook Rd Suite 160, Summit, NJ 07901 USA

**Keywords:** Hospital value proposition, NSQIP, PINPOINT, Intraoperative fluorescence imaging, Colorectal surgery, Anastomotic leak

## Abstract

**Background:**

The PILLAR II trial demonstrated PINPOINT is safe, feasible to use with no reported adverse events and resulted in no anastomotic leaks in patients who had a change in surgical plan based on PINPOINT’s intraoperative assessment of tissue perfusion during colorectal resection. Whether the cost savings associated with this reduction in anastomotic complications can offset the cost of investing in PINPOINT is unknown.

**Methods:**

We performed a retrospective analysis of all patients (*N* = 347) undergoing colectomy with primary anastomosis from January 2015 to April 2016. These patients were stratified based on whether fluorescence imaging was used intraoperatively. The clinical outcomes of these patients were then evaluated based on their development of an anastomotic leak or stricture. The direct hospital costs per case were then calculated, and the economic impact of using fluorescence imaging was examined to assess whether decreased direct costs would justify the initial expenditures to purchase new technology (PINPOINT System, NOVADAQ, Canada).

**Results:**

Fluorescence imaging in colorectal surgery using PINPOINT reduced the anastomotic failure rate in patients who underwent colon resection. The PINPOINT group (*n* = 238) had two (0.84%) anastomotic failures, while the non-PINPOINT group (*n* = 109) had six (5.5%) anastomotic failures. In the PINPOINT group, 11 (4.6%) patients had a change in the resection margin based on the results of the fluorescence imaging, and none of these patients experienced an anastomotic failure. Cost per case was less in the PINPOINT group secondary to fewer direct costs associated with complications.

**Conclusions:**

These results validate the findings of the PILLAR II trial and confirm the decrease in direct costs due to reduction in anastomotic failures as a result of using PINPOINT justified the expense of the new technology after just 143 cases.

The SPY System (NOVADAQ Technologies, Mississauga, Ontario, Canada) was first introduced over a decade ago and is widely used in open and minimally invasive surgery [1]. The clinical and economic benefits of SPY technology have been featured in numerous peer-reviewed publications [2–7]. SPY technology is available in a variety of platforms including the PINPOINT Endoscopic Fluorescence Imaging System.

PINPOINT allows for the real-time evaluation of vascular blood supply to various anatomic structures during laparoscopic surgical procedures. For example, PINPOINT can assess the microvascular blood flow to the colon both before resection and after reconstruction at the planned site of anastomotic creation [8]. This feature is clinically relevant for averting postoperative anastomotic failure, as it is often related to ischemia [9]. PINPOINT accomplishes this by enhancing the vascular evaluation at the time of selection of the site for colon transection and by allowing surgeons to appreciate the potential for a poorly perfused anastomosis. Most importantly, this informed evaluation enables surgeons to immediately make revisions while in the operating room in an effort to prevent anastomotic failure days later.

The PILLAR II study, a prospective, multi-center clinical trial, validates the PINPOINT system is a safe and feasible tool for intraoperative assessment of tissue perfusion during colorectal resection, and confirms that its use may lead to a decreased incidence of anastomotic leaks [10]. PILLAR II results show PINPOINT contributed to a change in surgical plan related to planned anastomosis in 11 (8%) patients. The overall anastomotic leak rate in the study was 1.4% (*N* = 2) with no leaks in the 11 patients who had a change in surgical plan based on intraoperative fluorescence imaging assessment with PINPOINT.

As the healthcare system continues to transition from a volume-based system to a value-based system where the quality of patient care and outcomes matter, it is increasingly important to carefully evaluate the cost-benefits of adopting a new technology, especially when costly. Understanding a novel technology’s clinical and economic benefits allows providers to more effectively and efficiently allocate their limited resources. We therefore performed a retrospective cost analysis to determine if the potential cost savings as a result of avoided anastomotic complications justified the acquisition of PINPOINT, a new technology to Overlook Medical Center (OMC).

## Methods

### Study design

This was a retrospective analysis (clinical and financial) of all patients undergoing colectomy with anastomosis over a 15-month period to assess for anastomotic failure (leak or stricture). Use of fluorescence technology was based on surgeon preference. The data were used to determine the postoperative incidence of and costs associated with anastomotic complications following colorectal surgery.

### Inclusion criteria

All patients undergoing an intestinal resection with a colonic or rectal anastomosis from January 2015 to April 2016 were included in this study.

### Exclusion criteria

Patients with a known allergy to iodide were excluded.

### Postoperative anastomotic outcomes

The anastomotic complications considered for this study were leaks and strictures. Both were captured by performing individual chart reviews on all colorectal cases to screen for these postoperative occurrences.

Leaks or disruption of the anastomosis was confirmed by radiographic imaging or by surgical intervention. Hospital chart reviews were performed to identify outcomes. The patient was tracked for postoperative interventions in the hospital data base after discharge.

Strictures were defined as anastomotic narrowing to ≤ 11 mm (identified endoscopically or on radiographic imaging) or narrowing that required endoscopic dilatation due to obstructive symptoms.

The postoperative period is defined as 30 days from the procedure date.

### Intraoperative measures

Three intraoperative outcomes were included in this study: a change in resection margin, revision of anastomosis, and additional resection margin.

Change in resection margin refers to the number of patients who were identified by PINPOINT to have insufficient perfusion of the bowel which was to be used for the anastomosis resulting in further resection.

Revision on anastomosis refers to the number of patients who were identified by PINPOINT to have insufficient perfusion of the bowel after the anastomosis was completed resulting in further resection.

Additional resection margin is the amount of intestine resected to obtain bowel margins, as assessed by PINPOINT, with appropriate perfusion to perform a reliable anastomosis.

### Direct costs

Direct costs were the average of the costs taken from the OMC data system and included supply costs, staff salaries, and operating room and hospital room charges. Total costs were calculated by multiplying average costs with incidence percentages based on the study results.

### Statistical analyses

The Chi-square test was used to compare categorical variables. Cost data were based on means. The null hypothesis is that there is no difference in anastomotic leaks with or without the use of PINPOINT. The alternative hypothesis is that anastomotic leaks are decreased when PINPOINT is used.

The study compared patient demographics to ensure both groups were similar and to identify variables that were associated with or contributed to anastomotic leaks. Consequently, a univariate or multivariate analysis was not performed.

## Results

### Patient population

This analysis included 347 patients treated for colorectal surgery at OMC from January 2015 to April 2016. Table 1 confirms the PINPOINT (*n* = 238) and non-PINPOINT (*n* = 109) groups were comparable across all variables except in transfusion percentage (16.4 vs. 8.3, *p* = 0.04), diverting ileostomy (5.5 vs. 13.8%, *p* = 0.008), cancer status (26.9 vs. 14.7%, *p* = 0.01), and operative approach (laparoscopic: 87 vs. 49.5%, *p* = 0001; open: 13 vs. 50.5, *p* = 0001).

**Table 1:** Patient demographics of PINPOINT and non-PINPOINT users

Demographics	PINPOINT (*N* = 238)	Non-PINPOINT (*N* = 109)	*P* value
Age (years), mean	62.4	60.8	0.30
Male sex (%)	52.9	45.8	0.25
BMI (kg/m^2^), mean	28.4	27.4	0.42
Albumin (g/dL), mean	3.6	3.4	0.08
Tobacco use (%)	16.8	14.7	0.75
Transfusion (%)	16.4	8.3	0.04
Diabetes (%)	2.5	5.5	0.20
ASA Class (%)			
I	12 (5%)	7 (6.4%)	0.60
II	152 (64%)	66 (60.5%)	0.47
III	67 (28%)	32 (29.4%)	0.70
IV	7 (3%)	4 (3.7%)	1.0
Diverting Ileostomy	13 (5.5%)	15 (13.8%)	0.008
Diagnosis			
Diverticular disease	80 (33.6%)	31 (28.4%)	0.38
Cancer	64 (26.9%)	16 (14.7%)	0.01
Polyp	66 (27.7%)	28 (25.7%)	0.79
Hartmann closure	21 (8.8%)	13 (12.0%)	0.44
Other (IBD, prolapse, volvulus, colonic inertia)	7 (3.0%)	21 (19.2%)	
Operative Approach			
Laparoscopic	207 (87%)	54 (49.5%)	0.0001
Open	31 (13%)	55 (50.5%)	0.0001

*BMI* Body mass index, *ASA* American Society of Anesthesiologists Physical Status Classification

### Outcomes

Table 2 shows the use of PINPOINT Fluorescence Imaging System in laparoscopic surgery at OMC from the beginning of 2015 which resulted in significant reductions in postoperative complications. 68.6% (238/347) of the patients were assessed with PINPOINT. There were no significant differences in abdominal abscess, wound infection, and ileus between the PINPOINT and non-PINPOINT study groups. However, there was a significantly higher incidence of anastomotic complications in non-PINPOINT users than in PINPOINT users, specifically anastomotic leakage (3.7 vs. 0.84%, *p* = 0.03) and anastomotic leakage and strictures in non-PINPOINT users (5.5 vs. 0.84%, *p* = 0.004).

**Table 2:** Postoperative morbidity by PINPOINT user status

Morbidity	PINPOINT (*N* = 238)	Non-PINPOINT (*N* = 109)	*P* value
Abdominal abscess, no. (%)	3 (1.3)	5 (5.0)	0.11
Wound infection, no. (%)	3 (1.3)	3 (2.8)	0.38
Ileus, no. (%)	22 (9.2)	13 (11.9)	0.44
Anastomotic leak, no. (%)	2 (0.84)	4 (3.7)	0.03
Anastomotic leak and strictures, no. (%)	2 (0.84)	6 (5.5)	0.004

Table 3 shows the intraoperative interventions administered as a result of the PINPOINT analysis. The use of PINPOINT resulted in a change in resection margin in 4.6% (11/238) of the patients and resulted in a revision of an already completed anastomosis in 0.4% (1/238) of the patients. The additional resection margin changed an average of 2.73 ± 1.6 cm. None of the patients who underwent an anastomotic revision or additional resection prior to anastomosis experienced a postoperative anastomotic leak or stricture [11].

**Table 3:** Intraoperative intervention resulting from PINPOINT analysis

Intraoperative intervention based upon PINPOINT	PINPOINT user (*N* = 238)
Change in resection margin, no. (%)	11 (4.6)
Revision of anastomosis, no. (%)	1 (0.4)
Additional resection margin, (cm), mean ± SD	2.73 ± 1.6

Table 4 compares the patient attributes of PINPOINT and non-PINPOINT users who experienced anastomotic leakage. The PINPOINT group consisted of an equal number of males and females with an average age of 70 years. The average age of the non-PINPOINT group comprised solely of males was slightly younger at 59.8 years. The non-PINPOINT users were also tobacco users compared to the PINPOINT users (25 vs. 0%). However, there were twice as many diabetics in the PINPOINT group compared to the non-PINPOINT group (50 vs. 25%). Both PINPOINT and non-PINPOINT groups were classified with a similar ASA physical status class (2.5 vs. 2.8) indicative of patients with mild systemic disease without substantive functional limitations.

**Table 4:** Demographic characteristics of patients who experienced anastomotic leakage by PINPOINT user status

Patient	PINPOINT (*N* = 2)	Non-PINPOINT (*N* = 4)	All leaks (*N* = 6)
Age, years, mean	70	59.8	63.2
Sex, % male	50%	100%	83%
BMI	31.8	30.8	31.1
Tobacco use, % smokers	0%	25%	17%
Albumin, g/dL	3.1	3.3	3.2
Diabetes	50%	25%	33%
ASA Class	2.5	2.8	2.7
Blood administered	50%	75%	67%
Operation, % right-sided anastomosis	100%	50%	67%
Operative approach, % laparoscopic	100%	75%	83%

*BMI* Body mass index, *ASA* American Society of Anesthesiologists Physical Status Classification

Table 5 examines the demographic characteristics of the six patients who experienced anastomotic leakage. One of the six patients affected by anastomotic leakage is female. The majority of patients are tobacco users and diabetic. The ASA physical status class ranged from 2.1 to 4. A level 4 indicates a patient with severe systematic disease that is life threatening.

**Table 5:** Demographic characteristics of individual patients who experienced anastomotic leakage

Patient	1	2	3	4	5	6
Age, years	72	68	60	46	78	55
Sex	F	M	M	M	M	M
BMI	35.8	27.7	38.9	22.5	24.6	37.3
Tobacco use	No	No	No	No	Yes	No
Albumin, g/dL	2.1	4	3.4	3.2	2.9	3.6
Diabetes	Yes	No	No	No	No	Yes
ASA Class	III	II	II	III	IV	II
Blood administered	Yes	No	No	Yes	Yes	Yes
Diagnosis	Cancer	Polyp	Diverticular disease, chronic	Diverticular disease, bleeding	Perforated viscus	Cancer
Operation	Extended right colectomy	Right colectomy	Left colectomy	Right colectomy	Extended right colectomy	Ultra low anterior resection
Operative approach	Lap	Lap	Lap	Lap	Open	Lap
PINPOINT Used	Yes	Yes	No	No	No	No

*BMI* Body mass index, *ASA* American Society of Anesthesiologists Physical Status Classification, *F* female, *M* male

### Costs

Table 6 shows the use of PINPOINT in patients undergoing major colon resections surgery which resulted in cost savings during the study period. Cost data were calculated per 100 patients using the PINPOINT user status percentage leak rates (3.7 vs. 0.8%) and the average cost per patient with and without a postoperative anastomotic leak ($60,625 vs. $14,375). Postoperative costs for non-PINPOINT users were 9.1% more than the average postoperative costs associated with PINPOINT users ($16,086 vs. $14,745). With the cost of the ICG dye factored in this results in an average cost savings of $1216 per case or to $121,600 in average cost savings for 100 cases.

**Table 6:** Postoperative costs associated with and avoided by using PINPOINT

PINPOINT user status	Average cost per case	Probability % of leakage rate	Average cost per case per 100 cases
Non-PINPOINT user			
With stricture and leak	$60,625	3.7%	$2243
Without stricture and leak	$14,375	96.3%	$13,843
Total			$16,086
PINPOINT user			
With stricture and leak	$60,625	0.8%	$485
Without stricture and leak	$14,375	99.2%	$14,260
PINPOINT Kit (ICG) cost			$125
Total			$14,870
Average cost savings per PINPOINT user per 100 cases			$1216

## Discussion

Patients who underwent PINPOINT intraoperative fluorescence imaging during their surgery experienced significantly lower anastomotic failure rates (Table 2) and cost less to treat (Table 6) compared to those who were not treated with PINPOINT. Anastomotic leaks and failures are expensive to treat [12] and were the primary drivers for the cost differential between the PINPOINT and non-PINPOINT patients. Therefore, the costs of the equipment and the costs of the disposables (Table 6) to perform PINPOINT intraoperative imaging are offset by the improved quality and subsequent cost savings associated with the use of the PINPOINT system after just 143 colon resections.

**Fig. 1:**
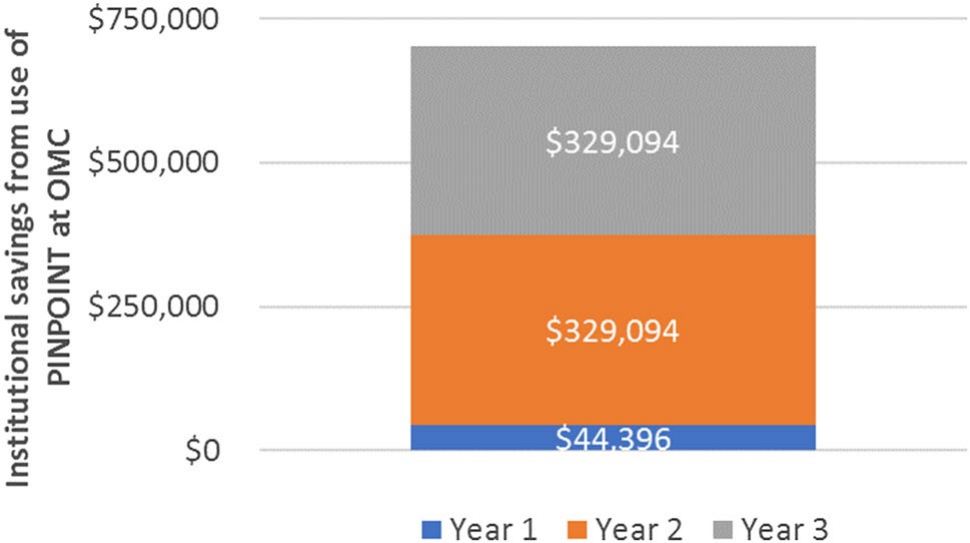
Projected cost savings associated with the use of PINPOINT at OMC

Over the next several years, with capital costs already incurred and all surgeons using the technology on every case, the cost savings associated with the use of PINPOINT and the related reductions in complications are projected to markedly increase as illustrated in Fig. 1.

Figure 1 shows a cost savings of $44,396 in the first year of PINPOINT’s implementation and projects a 641% increase in cost savings by the third year of its use due to the total adoption of the technology by all surgeons on staff.

The 11 cases that had a change in resection margin including one post anastomotic revision based on the use of fluorescence imaging demonstrate cases where anastomotic failure was presumably averted (Table 3). If these cases had not been revised, the anastomotic leaks and/or strictures that might have occurred would have led to an anastomotic failure rate of 5.5% in the PINPOINT group as well, mimicking the results seen in the non-PINPOINT group (Table 2) and being comparable to the national average of 6.2% [12]. The use of PINPOINT allowed the surgeons in our practice to proactively identify patients who were at high risk for anastomotic failure and act upon it at the index surgery.

The use of PINPOINT has had a significant positive impact on the clinical occurrence of anastomotic failure rates at OMC. Complications associated with anastomotic failure can be devastating and therefore, extremely expensive in terms of direct hospital costs. A retrospective study of 12,348 patients found total hospital costs per 1000 patients was 64.6% higher for patients with anastomotic leaks than for patients without anastomotic leaks ($72.9 million vs. $44.3 million) [12].

Fortunately, the value of the use of fluorescence imaging also includes intangible benefits that occur with the minimization of the complications associated with anastomotic failure. Patients in whom anastomotic leaks can be avoided experience a better quality of life as reflected in significantly higher physical, emotional, and social follow-up assessment scores than their counterparts who experienced anastomotic leaks [13].

In the modern era of medical reimbursement, value-based programs such as the Hospital Acquired Condition (HAC) Reduction Program, Readmission Reduction Program (RRP), and the Value-Based Purchasing (VBP) Programs have been implemented in U.S. hospitals as part of the initiative to move toward a value-based system [14]. Complications such as catheter-associated urinary tract infection (CAUTI), central line-associated blood stream infection (CLABSI), postoperative deep venous thrombosis/pulmonary embolus, Iatrogenic pneumothorax, and deep space surgical site infection (SSI) in colon surgery are all events which carry the potential for financial penalty of up to 6% of an institution’s base operating DRG payment [15]. This penalty percentage may increase in the future, making such complications even more expensive to institutions.

PINPOINT’s ability to improve patient quality care and outcomes is increasingly important for providers as well, as Centers for Medicare and Medicaid Services (CMS) continues to move toward a value-based payment model with its recent implementation of the Merit-Based Incentive Payment System (MIPS) [16–18]. Effective this year, CMS is collecting performance data on eligible physicians which will determine if providers receive a payment bonus or penalty based on their performance threshold score. By 2022, the maximum payment adjustment for a physician’s performance year will be ± 9%. The use of PINPOINT may help physicians meet their threshold score, as the MIPS performance categories are based on quality, cost, improvement activities, and advancing care information, all of which can be improved upon or demonstrated by the outcomes associated with the use of PINPOINT.

### Limitations

Strictures could not be adequately tracked because these were identified after discharge and could only be identified if patients were re-admitted to Overlook. It is possible more strictures were present but could not be identified because outpatient patient records were not available, and if strictured patients were done in an ambulatory surgical center, there would be no way to track this.

This is also a retrospective study of OMC, a single institution. Therefore, selection bias may be present, and the results of this study may not be generalizable to other facilities. A larger sample size and long-term follow-up would also improve the study design of this study.

## Conclusion

After the introduction of PINPOINT technology in December of 2014, there were both early and late adopters which enabled this comparative study. However, historical data at OMC using the National Surgical Quality Improvement Program (NSQIP) database confirmed our anastomotic leak rate to be 3.7%, equivalent to the non-PINPOINT cases during the study period.

In addition, further evaluation of these types of cases has shown continued good outcomes with the use of this technology. Through the end of 2016, PINPOINT technology has been used in over 450 cases with an anastomotic failure rate of 0.9%.

The basis of medical reimbursement is going to become more and more dependent on value rather than volume. Therefore, providers should consider implementing PINPOINT as a standard of care in colorectal surgeries to reduce the likelihood of complications and to alleviate institution costs and costs to patients who may experience poor postoperative outcomes such as anastomotic leaks. Such reduction in complications will also result in an as of yet unquantifiable increase in reimbursement based on the ever-growing volume of pay-for-performance variables.
